# Albuminuria Is Associated with Traditional Cardiovascular Risk Factors and Viral Load in HIV-Infected Patients in Rural South Africa

**DOI:** 10.1371/journal.pone.0136529

**Published:** 2015-08-26

**Authors:** G. Emerens Wensink, Annelot F. Schoffelen, Hugo A. Tempelman, Maarten B. Rookmaaker, Andy I. M. Hoepelman, Roos E. Barth

**Affiliations:** 1 Department of Internal Medicine & Infectious Diseases at the University Medical Centre in Utrecht, Utrecht, The Netherlands; 2 Ndlovu Care Group in Elandsdoorn, Limpopo, South Africa; 3 Department of Nephrology & Hypertension at the University Medical Centre in Utrecht, Utrecht, The Netherlands; University of Florida, UNITED STATES

## Abstract

**Context:**

As life expectancy improves among Human Immunodeficiency Virus (HIV) patients, renal and cardiovascular diseases are increasingly prevalent in this population. Renal and cardiovascular disease are mutual risk factors and are characterized by albuminuria. Understanding the interactions between HIV, cardiovascular risk factors and renal disease is the first step in tackling this new therapeutic frontier in HIV.

**Methods:**

In a rural primary health care centre, 903 HIV-infected adult patients were randomly selected and data on HIV-infection and cardiovascular risk factors were collected. Glomerular filtration rate (eGFR) was estimated. Albuminuria was defined as an Albumin-Creatinine-Ratio above 30 mg/g. Multivariate logistic regression analysis was used to analyse albuminuria and demographic, clinical and HIV-associated variables.

**Results:**

The study population consisted of 903 HIV-infected patients, with a median age of 40 years (Inter-Quartile Range (IQR) 34–48 years), and included 625 (69%) women. The median duration since HIV diagnosis was 26 months (IQR 12–58 months) and 787 (87%) received antiretroviral therapy. Thirty-six (4%) of the subjects were shown to have diabetes and 205 (23%) hypertension. In the cohort, 21% had albuminuria and 2% an eGFR <60 mL/min/1.73m^2^. Albuminuria was associated with hypertension (adjusted odds ratio (aOR) 1.59; 95% confidence interval (CI) 1.05–2.41; *p*<0.05), total cholesterol (aOR 1.31; 95% CI 1.11–1.54; *p*<0.05), eGFR (aOR 0.98; 95% CI 0.97–0.99; *p*<0.001) and detectable viral load (aOR 2.74; 95% CI 1.56–4.79; *p*<0.001). Hypertension was undertreated: 78% were not receiving treatment, while another 11% were inadequately treated. No patients were receiving lipid-lowering medication.

**Conclusion:**

Glomerular filtration rate was well conserved, while albuminuria was common amongst HIV-infected patients in rural South Africa. Both cardiovascular and HIV-specific variables were associated with albuminuria. Improved cardiovascular risk prevention as well as adequate virus suppression might be the key to escape the vicious circle of renal failure and cardiovascular disease and improve the long-term prognosis of HIV-infected patients.

## Introduction

The global impact of Human Immunodeficiency Virus (HIV) infection is immense. At the end of 2013, worldwide an estimated 35 million adults and children live with HIV and 1.5 million deaths were caused by Acquired Immune Deficiency Syndrome (AIDS) [1]. South Africa has one of the highest burdens of AIDS globally, with approximately 240,000 AIDS-related deaths in 2013 alone [2]. Fortunately, due to the enormous roll-out of antiretroviral therapy (ART), the incidence of HIV has stabilized and life expectancy of people living with HIV has improved tremendously [3]. HIV is now considered a chronic disease and its prevalence has subsequently increased. With an aging population of people living with HIV, the focus of today’s HIV care is shifting from treating acute illnesses caused by opportunistic infections towards reducing chronic comorbidities [3].

Cardiovascular and renal disease are important co-morbid conditions among patients with HIV, including patients from sub-Saharan Africa (SSA) [4]. Several factors predispose HIV-infected patients to developing renal disease. Factors directly associated with HIV infection include an increased susceptibility for renal infections, direct renal damage through HIV-associated nephropathy (HIVAN) and anti-HIV immune complex mediated glomerulonephritis (HIVICK) [5]. Nephrotoxicity can result from HIV-associated treatments, such as antimycotic agents, and ART, such as tenofovir and indinavir [6,7]. The increased prevalence of traditional cardiovascular risk factors seen in HIV-infected patients predisposes these patients even further to developing kidney damage [8,9]. Furthermore, African race is associated with a higher risk of hypertensive kidney disease and HIVAN, as well as a more aggressive course of kidney disease [10–12]. In addition to the burden of kidney disease, the prevalence of cardiovascular disease (CVD) is increasing in SSA, as well as among HIV-positive patients [13–15]. Among the general population in SSA, HIV is the leading overall cause of death, while CVD is second [16]. A vicious circle ensues where CVD causes kidney damage, which in turn worsens cardiovascular risk [17–19].

Albuminuria characterizes this vicious cardio-vascular-renal circle and is a marker for both renal disease and a prognostic marker for cardiovascular risk in HIV-positive patients [20,21]. HIV-infected patients with albuminuria are significantly more likely to develop a decreased glomerular filtration rate (GFR) than those without [20]. In addition, albuminuria is associated with a higher risk of developing CVD and a higher all-cause mortality rate in HIV-positive patients [21–25]. An increased risk for experiencing an atherosclerotic cardiovascular event (CVE) and heart failure have been described in HIV-infected patients with albuminuria, as compared to those without [22].

Albuminuria results from an altered balance between glomerular filtration and tubular resorption, both of which may be altered by diabetes, hypertension, HIV infection and its potentially nephrotoxic medication [26,27]. The association between clinical variables with albuminuria has been studied in different populations of HIV-positive patients [20,28–33]. Both cardiovascular and HIV-associated risk factors, such as diabetes, hypertension, viral load and type of ART regimen, were found to be linked to albuminuria [20,28–33]. Although associations are known, the exact mechanism for the increased CVD risk in HIV-infected patients with albuminuria remains unclear. Possibly, associations can be explained by a common pathophysiologic process, such as endothelial dysfunction or chronic low-grade inflammation [34]. HIV infection is expected to cause chronic inflammation and may thus play a role in the increased risk [35]. ART can act additionally in two ways, decreasing inflammation through suppressing HIV viral load, as well as increasing inflammation through its toxic side effects.

To our knowledge, the interaction between impaired GFR, albuminuria and cardiovascular and HIV-associated determinants has not yet been studied amongst HIV-positive patients in SSA. However, data from Western countries cannot simply be extrapolated to SSA, the most impacted area by the HIV pandemic, for several reasons. First, chronic kidney disease is three to four times more prevalent in this area and CVD has vastly increased [16,36]. Secondly, the type and nature of kidney disease amongst HIV-infected patients in SSA differs, with generally a more aggressive course of HIVAN in patients with African ancestry [12]. Thirdly, the decreased access to, and often delayed start of, ART in SSA patients results in a higher risk of developing HIV-associated renal disease [6,37]. Early identification of patients at increased risk of developing renal disease is especially important in SSA. Patients often cannot afford renal replacement therapy, making renal failure a terminal condition [38]. Sub-Saharan Africans form not only an important population to investigate the relation between HIV, renal disease and CVD, but due to the high incidence and the limited access to dialysis and transplantation, it is also the population that would benefit most from preventive measures to attenuate progression of renal and vascular disease.

The current study aimed to provide epidemiological data on the prevalence of decreased GFR and albuminuria. Additionally, the study aimed to describe the association of cardiovascular and HIV-related factors with albuminuria among HIV-positive patients in a rural population in South Africa as an essential first step towards the identification of factors amenable to therapeutic intervention. Thus aiding in the development of treatments to face the new frontiers in the care for HIV infected individuals.

## Methods

### Setting and Study Population

The cohort consisted of 903 adult patients (aged 18–74 years) from the Ndlovu Medical Centre (NMC) in South Africa. NMC provides primary health care in Elandsdoorn, a rural area in Limpopo, South Africa (www.ndlovucaregroup.com). The clinic provides care to a population of approximately 120 000–140 000 patients in the area. A fully funded ART program is available since 2003, including HIV treatment and testing for approximately 3 600 HIV-positive patients. A previous study demonstrated that HIV in this region is generally of the HIV-1 subtype-C origin [39].

### Data Collection

Known adult HIV-infected patients visiting the outpatient ward for routine clinical care were randomly selected from the waiting queue by research personnel through blind selection of the medical file. Patients were eligible regardless of severity of disease or comorbidities and were included after obtaining written informed consent. Patients without a urine sample available (n = 3) were excluded from the study. Data collection took place from May 1^st^ 2013—December 9^th^ 2013, by trained research personnel using a standardized protocol. In a one-time clinical visit, weight, height, waist circumference, pulse rate and blood pressure were examined according to standard procedure. Blood pressure was measured manually using a sphygmomanometer after ten minutes of rest in a sitting position, using Korotkoff tones to identify the systolic and diastolic pressures. Three separate blood pressure measurements were made on the left and the right arm, with the third measurement on the arm with the highest blood pressure, where the average of the three was recorded. When indicated, due to an inappropriate blood cuff size fit, a larger blood cuff size was used. The Body Mass Index (BMI) was calculated as the weight in kilograms divided by the square of height in meters. A baseline questionnaire was completed to obtain demographic information and additional information concerning self-reported alcohol and tobacco use, family history of CVD, self-reported history of CVD, diabetes and medication use. Venous blood was collected into ethylenediaminetetraacetic acid (EDTA) and serum separating tube (SST) tubes and one random sample of urine was obtained. The Dade Dimension (Siemens, Germany) diagnostic analyzer was used to determine the values of serum creatinine, alanine aminotransferase (ALT), total cholesterol, high density lipoprotein (HDL) cholesterol, triglycerides, glycated hemoglobin (HbA1c) and urine ACR using commercial Siemens kits in the laboratory (Toga laboratories, accreditation by the South African National Accreditation System (SANAS)). Plasma HIV-1 viral loads (System 340 bDNA analyzer, Bayer, Germany) and CD4 cell counts (fc500 MPL Coulter analyzer, Beckman Coulter, United States) were determined. Medical history was obtained from the medical file, including information on date of HIV-infection diagnosis, start date of ART, current ART-regimen, most recent CD4 cell count and most recent HIV-1 viral load (both less than 1 year old). Any abnormalities found were reported back to the treating clinician for further follow-up. Routine clinical management was not interrupted for the study. Ethical approval was received from the Research Ethics Committee of the Faculty of Health Sciences from the University of Pretoria. Research was conducted according to the Helsinki Declaration of 1975.

### Statistical Analysis

Estimated glomerular filtration rate (eGFR) and albuminuria were the primary outcome variables. eGFR was calculated according to the 2009 Chronic Kidney Disease Epidemiology Collaboration (CKD-EPI) formula and the Modification of Diet in Renal Disease (MDRD) formula [40,41]. eGFR was categorized in three categories: eGFR ≥ 90 mL/min/1.73m^2^, eGFR 60–90 mL/min/1.73m^2^ and eGFR< 60 mL/min/1.73m^2^ [41]. Albuminuria was categorized using the urinary Albumin-Creatinine-Ratio (ACR) as moderately increased (ACR 30–300 mg/g) or severely increased (ACR > 300 mg/g) [41].

Demographic, traditional cardiovascular risk factors and HIV-specific covariates were analysed for a possible association with the primary outcome. Demographic variables included age and gender. Traditional CVD risk factors included diabetes mellitus (use of diabetic medication or HbA1c > 6.5% [42,43]), hypertension (defined as diastolic blood pressure ≥ 90 mmHg and/ or systolic blood pressure ≥ 140 mmHg or use of antihypertensive medication) and the lipid profile. Total cholesterol, HDL cholesterol and triglycerides were measured and low-density lipoprotein (LDL) was determined using the Friedewald formula [44]. Total cholesterol, HDL cholesterol and triglycerides were analysed as continuous variables, without cut-off values. Obesity was defined as a BMI above 30 kg/m^2^ [45]. Waist circumference was measured and defined as enlarged in case of a circumference >94 cm for men and >80 cm for women [45]. A positive family history for CVE was defined as having at least one first degree relative (parent or sibling) who had had a heart attack or stroke before the age of 60 years. A history of CVE was defined as a self-reported stroke or heart attack or symptoms matching a stroke or heart attack. The HIV viral load was categorized as undetectable VL (VL<50 copies/mL) and elevated VL (VL≥50 copies/mL, including all patients not currently on ART, whose viral loads were unknown, but assumed to be over 50 copies/mL). The total duration of ART exposure, duration since Voluntary Counselling and Testing (VCT) and current exposure to tenofovir, abacavir, protease-inhibitor (PI) or non-nucleoside reverse-transcrip!ase inhibitor (NNRTI) were obtained from medical records.

The above-mentioned variables were examined for their association with albuminuria using regression models. Initially, each variable was analysed in univariate analysis using either the Mann-Whitney U test for continuous variables or the Chi-squared test or Fisher’s Exact Test for categorical variables. The variables used in the regression models were either associated in univariate analysis (*p*<0.10) or known to be linked to the outcome in literature. In case of collinearity between variables (calculated by a Variance Inflation Factor ≥ 5), only the variable with the least missing data was included. A forced-entry binary logistic regression model was used. Adjusted Odds Ratios (aOR) for the variables were calculated, where a *p*-value below 0.05 was considered statistically significant.

Viral load was not included in regression analysis of the whole cohort, due to a high proportion of missing viral load data for patients who had been receiving ART for less than 6 months. A separate logistic regression analysis was performed including only those patients who had been receiving ART for more than 6 months, in which the association between viral load and albuminuria was analysed as well.

All statistical analyses were performed using IBM SPSS Statistics software, version 22.

## Results

The characteristics of the 903 patients included in the cohort are presented in Table 1. The median age was 40 years (Inter-Quartile Range (IQR) 34–48 years) and female gender was predominant (69%). Similar cohort characteristics were obtained for the 656 patients (73%) included in the subgroup analysis, containing only those patients who had been on ART for at least six months (S1 Table).

**Table 1:** Characteristics of the study population.

Variable	ACR ≤30 mg/g	ACR >30mg/g	Total
**Demographic factors**			
N (%)	715 (79)	188 (21)	903
Age in years	39 [34–46]	43 [36–51]	40 [34–48]
Gender Female	492 (69)	133 (71)	625 (69)
**HIV status**			
Months since positive HIV test	27 [13–60]	24 [7–53]	26 [12–58]
Most recent CD4 cell count (cells/mm^3^)	406 [212–610]	327 [133–597]	387 [204–603]
HIV-1 VL (copies/mL)			
*HIV-1 VL <50*	-	-	*524 (58)*
*HIV-1 VL 50–999*	-	-	*60 (7)*
*HIV-1 VL ≥1000*	-	-	*56 (6)*
*Missing HIV-1 VL*	-	-	*263 (29)*
On ART	622 (87)	165 (88)	787 (87)
*NNRTI regimen (% of patients on ART)*	*577 (93)*	*154 (93)*	*731 (93)*
*PI-based regimen*	*45 (7)*	*11 (7)*	*56 (7)*
*Current Tenofovir Exposure*	*490 (79)*	*122 (74)*	*612 (78)*
*Current Abacavir Exposure*	*6 (1)*	*7 (4)*	*13 (2)*
*Duration on ART in months*	*25 [11–52]*	*20 [5–47]*	*24 [10–51]*
**Cardiovascular risk factors**			
BMI>30 kg/m^2^	135 (19)	26 (14)	161 (18)
Large Waist Circumference ^a^	329 (46)	78 (42)	407 (45)
Current smoker	105 (15)	25 (13)	130 (14)
Diabetes Mellitus ^b^	20 (3)	16 (9)	36 (4)
Total cholesterol (mmol/L)	4.30 [3.60–4.90]	4.60 [4.00–5.40]	4.40 [3.70–5.00]
LDL cholesterol (mmol/L)	2.47 [1.88–2.96]	2.61 [2.00–3.33]	2.49 [1.90–3.00]
Hypertension ^c^	142 (20)	63 (33)	205 (23)
Family History	39 (6)	12 (7)	51 (6)
Previous CVE	17 (2)	3 (2)	20 (2)
**Laboratory values**			
eGFR _CKD-EPI_ (mL/min/1.73m^2^)	122.5 [107.7–133.7]	111.8 [89.8–129.9]	119.9 [103.9–133.0]
*eGFR< 60*	*1 (0)*	*17 (9)*	*18 (2)*
*eGFR 60–90*	*57 (8)*	*30 (16)*	*87 (10)*
*eGFR ≥ 90*	*657 (92)*	*141 (75)*	*798 (88)*
eGFR _MDRD_ (mL/min/1.73m^2^)	114.2 [98.2–132.4]	103.1 [84.7–123.9]	112.9 [95.7–131.9]
*eGFR< 60*	*3 (0)*	*17 (9)*	*20 (2)*
*eGFR 60–90*	*82 (12)*	*45 (24)*	*127 (14)*
*eGFR ≥ 90*	*630 (88)*	*126 (67)*	*756 (84)*
Creatinine_serum_ (umol/L)	66 [57–76]	70 [58–86]	66 [57–78]
ALT (U/L)	23 [17–33]	24 [19–36]	23 [17–33]

Data are given as number (%) or median [Inter-Quartile Range].

^a^ Large waist circumference: >94 cm men or > 80 cm women;

^b^ Diabetes mellitus: HbA_1_c > 6.5% or use of diabetes medication;

^c^ Hypertension: Systolic blood pressure ≥ 140 mmHg, diastolic blood pressure ≥ 90 mmHg or use of antihypertensive medication.

ACR = Albumine—Creatinine Ratio; ALT = alanine aminotransferase (mmol/l); ART = anti-retroviral treatment; BMI = Body Mass Index; CVE = cardiovascular event; CKD-EPI = Chronic Kidney Disease—Epidemiology; eGFR = estimated glomerular filtration rate; HIV = Human Immunodeficiency Virus; LDL = Low-density lipoprotein; MDRD = Modification of Diet in Renal Disease; NNRTI = Non-nucleoside reverse-transcriptase inhibitors; PI-based = protease inhibitor-based; VL = viral load.

Moderately increased albuminuria (ACR 30–299 mg/g) was present in 20% (181/903) of patients and severely increased albuminuria (ACR >300 mg/g) in 1% (7/903). Characteristics of the patients with severely increased albuminuria are described in the supporting information (S3 Table). In the whole cohort, the vast majority of patients had a normal eGFR, with only 18 patients (2%) having an eGFR<60 mL/min/1.73m^2^, and 87 patients (10%) having an eGFR 60–90 mL/min/1.73m^2^. Since decreased eGFR was rare, the power to compare the distribution of characteristics among subgroups was insufficient and further analysis of the relation between decreased eGFR and covariates could not be performed. The results for the prevalence of impaired GFR were similar for both the MDRD and the CKD-EPI calculated eGFR (data not shown). Unless otherwise noted, the CKD-EPI eGFR will be recorded.

Albuminuria was linked with a higher frequency of diabetes, hypertension, higher total cholesterol and decreased eGFR. Of the 20 patients with an eGFR<60 mL/min/1.73m^2^, all but three (85%) had albuminuria. After adjustment for confounding variables using regression analysis, hypertension (aOR 1.59; 95% CI 1.05–2.41; *p*<0.05) and total cholesterol (aOR 1.31; 95% CI 1.11–1.54; *p*<0.05) remained independently associated with albuminuria in the whole cohort (Table 2). Furthermore, higher eGFR, was significantly linked with a lower prevalence of albuminuria (aOR 0.98; 95% CI 0.97–0.99; *p*<0.001), for both the CKD-EPI and MDRD formula. Other variables were not related to albuminuria after adjustment for covariates. Similar to the observation in the full cohort, hypertension, total cholesterol and eGFR (both if calculated with the CKD-EPI equation and the MDRD formula) were also independently linked with albuminuria in the smaller cohort including only patients on ART for at least six months (S2 Table).

**Table 2:** Variables associated with albuminuria ^a^ in univariate and multivariate analysis in an unselected group of HIV-infected patients.

Variable	Univariate *p*-value	Multivariate aOR (95% CI)	Multivariate *p*-value
**Demographic factors**			
Age in years	0.001*	0.990 (0.970–1.012)	0.37
Gender Female	0.61	-	-
**HIV status**			
Months since positive HIV test	0.06 ^+^	0.997 (0.990–1.003)	0.31
Most recent CD4 cell count (cells/mm^3^)	0.01 *	0.999 (0.999–1.000)	0.07
HIV-1 VL copies/mL	-	-	-
On ART	0.78	-	-
*NNRTI regimen (% of ART)*	*0*.*71*	*1*.*075 (0*.*614–1*.*880)*	*0*.*80*
*PI-based regimen*	*0*.*82*	-	-
*Current Tenofovir Exposure*	*0*.*34*	*0*.*831 (0*.*515–1*.*342)*	*0*.*45*
*Current Abacavir Exposure*	*0.01* *	*1*.*674 (0*.*447–6*.*276)*	*0*.*44*
Duration on ART in months (if started with ART previously)	0.02*	^y^	-
**Cardiovascular risk factors**			
BMI>30 kg/m^2^	0.11	-	-
Large Waist Circumference ^b^	0.29	-	-
Current smoker	0.63	-	-
Diabetes Mellitus ^c^	<0.001*	2.047 (0.965–4.343)	0.06
Total cholesterol (mmol/L)	<0.001*	1.308 (1.111–1.539)	<0.001*
LDL cholesterol (mmol/L)	0.006*	^Y^	-
Hypertension ^d^	<0.001*	1.592 (1.050–2.415)	0.03*
Family History	0.57	-	-
Previous CVE	0.78	-	-
**Laboratory values**			
eGFR _CKD-EPI_ (mL/min/1.73m^2^)	<0.001*	0.978 (0.970–0.987)	<0.001*
ALT (U/L)	0.09^+^	1.001 (0.995–1.007)	0.72

Included subjects in multivariate analysis: 865. Number of subjects with albuminuria: 174.

*p-values are significant (p<0.05);

^+^ p-values between 0.05 and 0.10;

^Y^ Excluded due to collinearity with another variable.

^a^ Albuminuria: ACR>30 mg/g;

^b^ Large waist circumference: >94 cm men or > 80 cm women;

^c^ Diabetes mellitus: HbA_1_c > 6.5% or use of diabetes medication;

^d^ Hypertension: Systolic blood pressure ≥ 140 mmHg, diastolic blood pressure ≥ 90 mmHg or use of antihypertensive medication.

ACR = Albumine—Creatinine Ratio; ALT = alanine aminotransferase (mmol/l); aOR = adjusted odds ratio; ART = anti-retroviral treatment; BMI = Body Mass Index; 95% CI = 95% Confidence Interval; CKD-EPI = Chronic Kidney Disease—Epidemiology; CVE = cardiovascular event; eGFR = estimated glomerular filtration rate; HIV = Human Immunodeficiency Virus; LDL = Low-density lipoprotein; MDRD = Modification of Diet in Renal Disease; NNRTI = Non-nucleoside reverse-transcriptase inhibitors; PI-based = protease inhibitor-based; VL = viral load.

**Fig 1:**
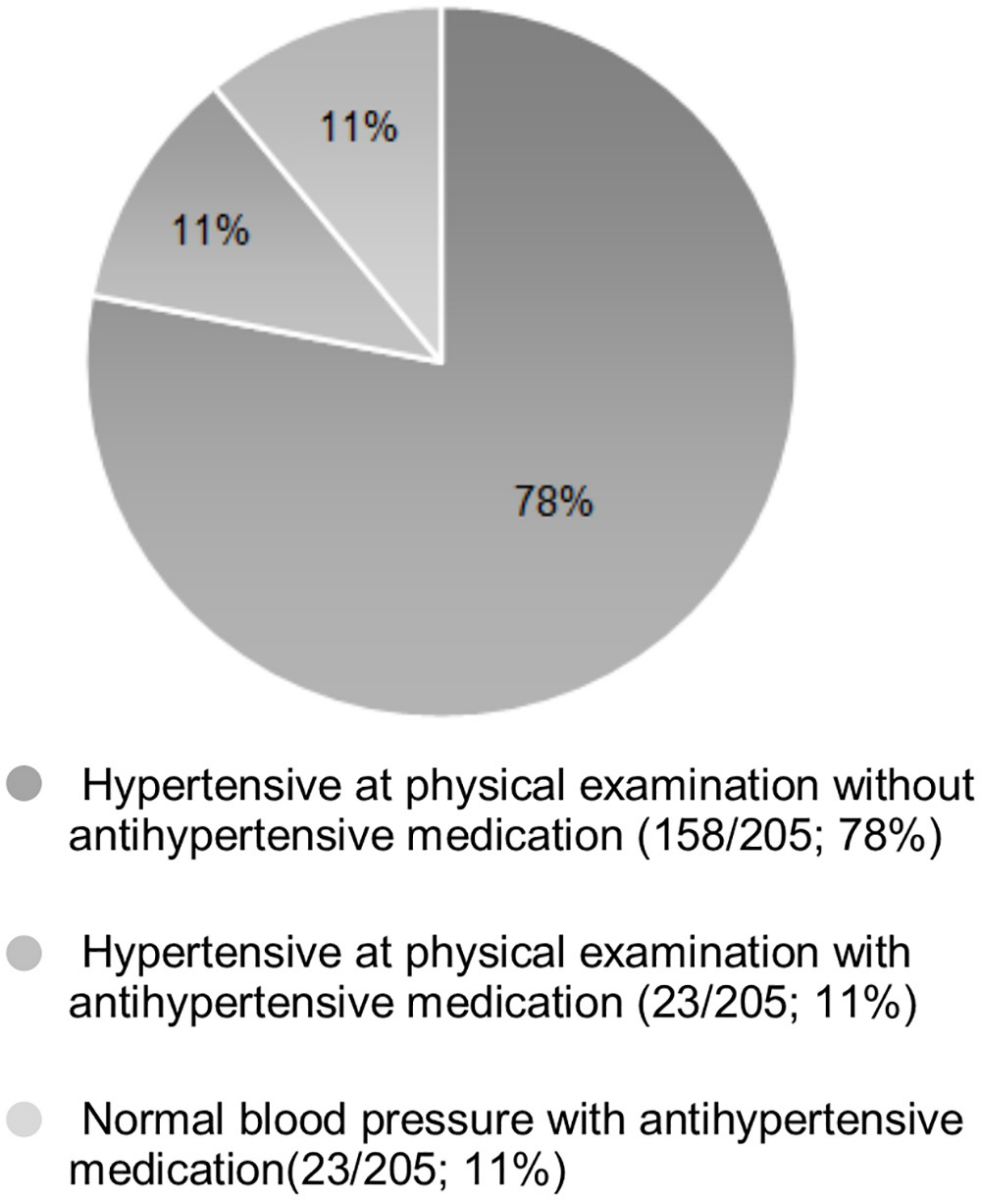
Hypertensive Control in HIV-positive patients (N = 205). Included subjects from the cohort of 903 HIV-positive patients: 205 patients, previously diagnosed with hypertension or in whom hypertension was newly diagnosed. Hypertensive = systolic blood pressure ≥140 mmHg and/or diastolic blood pressure ≥90 mmHg.

**Fig 2:**
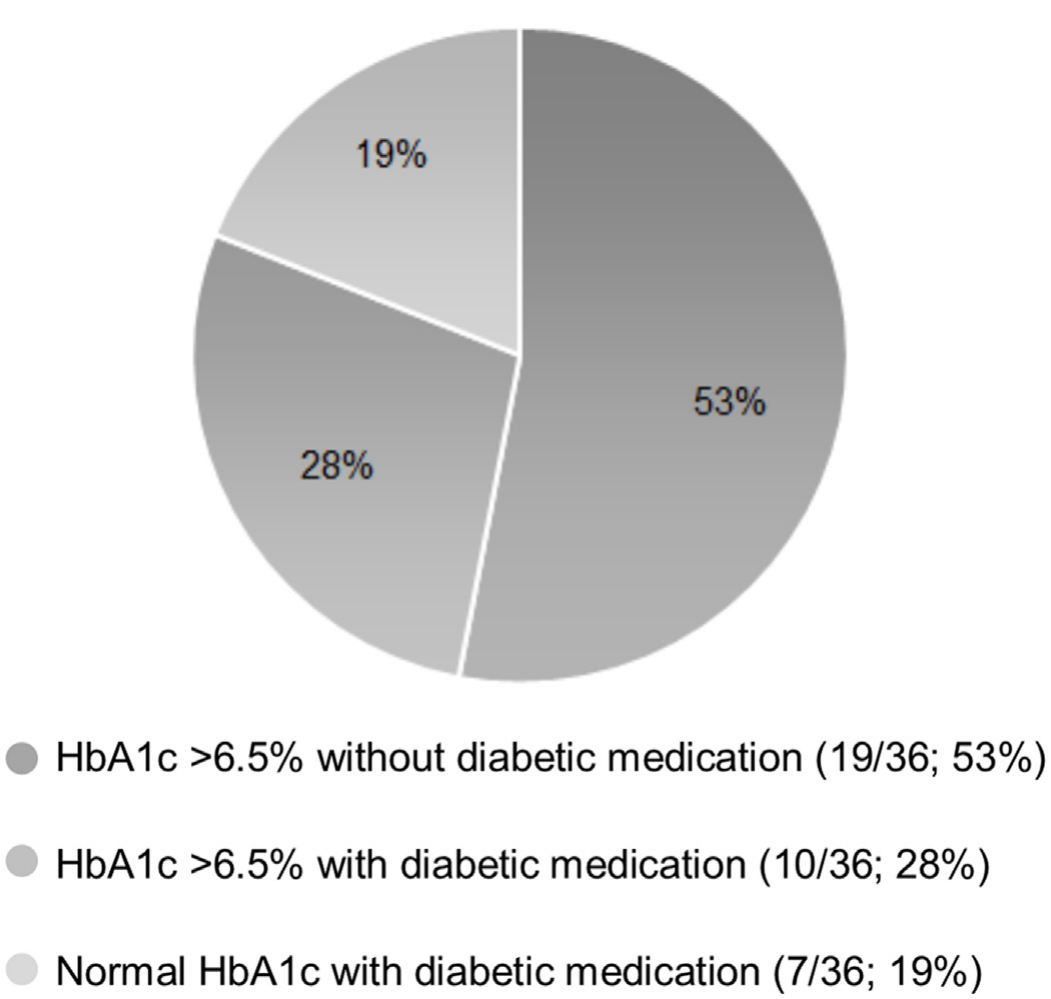
Diabetes Control in HIV-positive patients (N = 36). Included subjects from the cohort of 903 HIV-positive patients: 36 patients, previously diagnosed with diabetes or in whom a raised HbA1c was measured. Raised HbA1c = HbA1c > 6.5%.

Some cardiovascular risk factors were highly prevalent in the cohort. Obesity and hypertension were each observed in almost a fifth of patients, while diabetes was present in 4% of patients. Among study subjects, effective regulation of hypertension and diabetes was low. In hypertensive patients, 78% (159/205) did not receive any antihypertensive medication while an additional 11% (23/205) were inadequately regulated since they were still hypertensive despite being on antihypertensive therapy (Fig 1). Among diabetics, inadequately treated diabetes (HbA1c > 6.5%) was also prevalent (Fig 2). Of the 36 diabetic patients, 53% (19/36) were not receiving antidiabetic medication, while 28% (10/36) were inadequately regulated despite receiving treatment (Fig 2). None of the patients in the cohort were receiving lipid-lowering therapy, even though 25% of the patients had a total cholesterol above 5 mmol/L (Table 1).

Of the total group, the vast majority were receiving ART (87%), with 67% (524/787) having an undetectable viral load (HIV RNA <50 copies/mL), while only 7% (56/787) of patients showed clear virological failure (HIV RNA >1000 copies/mL). A viral load was not available for 263 patients (29%), being mostly ART-naïve patients or patients on ART for less than 6 months before inclusion in the study. The median CD4 cell count was 387 cells/mm^3^. Of all patients receiving ART, almost 80% was exposed to tenofovir at the moment of inclusion in the study, while 7% were on a PI-based regimen (not including indinavir).

Patients with albuminuria had a lower CD4 cell count versus those without albuminuria (median 327 cells/mm^3^ versus 406 cells/mm^3^, *p*<0.05) as demonstrated in Table 1 and Table 2. In the selection of patients receiving ART for more than 6 months, patients with albuminuria were also more likely to have a detectable viral load than patients without albuminuria (25% versus 15%, *p*<0.05; S2 Table). After adjustment for confounding variables, a detectable viral load was the only HIV-related factor that was associated with albuminuria (analysis performed including only patients on ART for at least six months (S2 Table)). The odds of having albuminuria was 2.74 (95% CI 1.56–4.79; *p*<0.001) times higher if a detectable viral load was present.

## Discussion

In this cohort of rural South African HIV-infected patients, albuminuria was present in 20% of patients, while eGFR below 60 mL/min/1.73m^2^ was observed in only 2% of all patients. Thus, glomerular filtration rate seems well conserved while albuminuria was prominently present. Both traditional risk factors eGFR, hypertension and total cholesterol, as well as HIV viral load, were independently associated with microalbuminuria.

The large number of patients with albuminuria is worrisome, since albuminuria both signals underlying renal disease and is related to a worse clinical outcome in HIV-infected patients [21–23]. The high frequency of albuminuria in HIV-positive African residents may indicate an increased risk of developing renal disease and cardiovascular events for these patients.

Reported albuminuria in similar cohorts of HIV-infected patients in SSA is reflective of our results, where albuminuria was observed among 18.5% to 36% of treatment-naïve and ART patients [46,47]. Similarly, our results are in line with previous South African studies where 0.7–6% patients had an eGFR below 60 mL/min/1.73m^2^ and 7.7–15.5% of patients had an eGFR below 90 mL/min/1.73m^2^ [9,48,49].

Published studies support the association between hypertension and eGFR with albuminuria in HIV-positive patients [28–33]. However, factors associated with albuminuria were not identical for each study, which may be due to a variation in the ethnical and demographic composition of included populations, such as Asian versus Western study groups, or differences in the pre-existing cardiovascular risk profile, for example inclusion of only diabetic patients.

This study is the first to demonstrate an independent association of total cholesterol with moderately increased albuminuria. However, an association with severely increased albuminuria has been described [20,32]. A lack of standard cut-off values used to define dyslipidemia may explain the different findings. There may also be confounding by ART regimen type, which is known to affect a patients’ lipid profile [50]. Nonetheless, the results in our study remained significant after adjusting for ART regimen type.

The association between traditional cardiovascular risk factors and albuminuria in HIV-infected patients reflects the dangerous interplay between CVD and renal dysfunction [17–19]. Our results do not convey causality. It would be interesting to know which is more effective in lowering the risk of cardiovascular and renal disease: lowering cholesterol levels using statins, adequately treating hypertension, or decreasing albuminuria with appropriate medication, such as Angiotensin I converting enzyme (ACE) inhibitors.

In the current study, no patients were receiving lipid-lowering therapy and the vast majority of hypertensive patients did not receive adequate antihypertensive treatment. As such, detecting and treating both dyslipidemia and hypertension may be an important target for therapeutic interventions to prevent future albuminuria and cardiovascular events. Financial constraints are likely to partly explain the low treatment rates for these cardiovacular risk factors, as cardiovascular preventive treatments are not generally provided for free, in contrast to ART and antituberculous treatment, which are being funded by governmental treatment programs.

Interestingly, only viral load—but neither low CD4 cell count nor ART regimen type or duration—was identified as an HIV-related factor associated with albuminuria. Therefore, the direct effect of HIV viremia on the kidneys seems to contribute more to the development of albuminuria than opportunistic infections (related to CD4 count) or nephrotoxic effects of ART in use in this population. HIVAN, a form of direct renal damage caused by HIV, has previously been shown to be strongly associated with the African race [11], which may partly explain the association between viral load and albuminuria in this study. An undetectable viral load decreases the likelihood of the presence of HIV-related nephropathies, as seen in studies examining renal biopsies [5,51,52]. Fortunately, a majority of HIV-positive patients in South Africa are successfully being treated, thereby reducing the numbers of patients with a detectable viral load and decreasing the nephrotoxic effect of HIV-viremia [3].

One disputed factor concerning renal disease in HIV-infected patients is tenofovir exposure. Tenofovir causes various degrees of proximal tubule dysfunction, from euglycemic glycosuria, phosphaturia or amino aciduria to an overt Fanconi syndrome [53]. The majority of the patients in the study were treated with tenofovir. However, the current study did not find an association between current tenofovir exposure and albuminuria, but it is not fully conclusive, since there are no recorded data concerning previous tenofovir exposure. As such, confounding due to previous exposure cannot be excluded. Additionally, tenofovir-associated proximal renal tubular dysfunction results in predominantly non-albumin proteinuria and is best detected using retinol-binding protein excretion [54,55]. Given that this study examined eGFR and albuminuria, without measuring hypophosphatemia, glycosuria or total urine protein levels, mild forms of tenofovir-associated nefrotoxicity may have been missed. No patients were treated with indinavir, which has been associated with decreased renal function in previous literature as well [56,57].

Some limitations of this study deserve attention. The current report concerns only observational data, due to the descriptive cross-sectional design of the study, and causality cannot be demonstrated. However, our study forms a solid base to design intervention studies to address causality and therapeutic efficacy. The outcome parameter was limited to albuminuria, as a one-time measurement, which may include transient albuminuria. In future studies, more specific markers for CVD, like pulse wave velocity or carotid intima media thickness, or for renal disease such as tubular damage markers and persistent albuminuria, should be assessed. Ideally, hard end points like cardiovascular events or end-stage renal disease should be included. Potential confounding from unmeasured variables, such as previous tenofovir exposure, opportunistic infections or urinary tract infections, could not be excluded. Our study estimated GFR using various formulae, instead of direct measurement. None of the formulae have been validated in HIV-positive patients, where weight may vary due to the clinical effects of HIV-infection. However our study population had a normal weight distribution, so the expected effect on the estimated GFR should be minimal [58].

In conclusion, in this large South African cohort study, glomerular filtration rate was well conserved while albuminuria was common in HIV-infected patients. This is the first known study to describe potentially modifiable factors in HIV-infected patients in SSA prone to develop cardiovascular and renal disease, reflected by an increased presence of albuminuria. The traditional cardiovascular risk factors eGFR, hypertension, and total cholesterol, as well as HIV viral load, were independently associated with albuminuria in these patients. Many patients were not adequately treated for hypertension and dyslipidemia, both of which were associated with albuminuria. Although no causality was demonstrated in our study, blood pressure, cholesterol and HIV viral load appear to be promising therapeutic targets to meet the future challenges of the increasing population of patients infected with HIV. A reorganisation of financial resources should be taken into consideration to support such a change in chronic health care delivery.

## Supporting Information

S1 Table Characteristics of the HIV-infected patients on ART for at least 6 months. (DOC)

S2 Table Variables associated with albuminuria in univariate and multivariate analysis in HIV-infected patients on ART treatment for > 6 months. (DOC)

S3 Table Characteristics of the patients with severely increased albuminuria (ACR>300 mg/g). (DOCX)
