# Supplementary material for: Albuminuria Is Associated with Traditional Cardiovascular Risk Factors and Viral Load in HIV-Infected Patients in Rural South Africa
Source: PLoS One. 2015 Aug 26;10(8):e0136529. doi: 10.1371/journal.pone.0136529 (PMC4550462; doi:10.1371/journal.pone.0136529)
Supplement: S1 Table — (DOC) [file pone.0136529.s001.doc]

**S1 Table. Characteristics of the HIV-infected patients on ART for at least 6 months**

| **Variable** | **ACR ≤30 mg/g** | **ACR >30mg/g** | **Total** |  |
| --- | --- | --- | --- | --- |
| N (%) | 536 (82) | 120 (18) | 656 (100) |  |
| **Demographic factors** | | | |  |
| Age (years) | 40 [34 - 46] | 43 [37 - 51] | 40 [35 - 47] |  |
| Gender Female | 376 (70) | 92 (77) | 468 (71) |  |
| **HIV status** | | | |  |
| Months since positive HIV test | 37 [19 - 68] | 40 [21 - 65] | 37 [19 - 67] |  |
| Most recent CD4 cell count (cells/mm^3^) | 440 [267 - 654] | 403 [232 - 651] | 440 [261 – 654] |  |
| HIV-1 VL (copies/mL) | - | - | - |  |
| *HIV-1 VL <50* | *429 (80)* | *82 (68)* | *511 (78)* |  |
| *HIV-1 VL 50 - 999* | *45 (9)* | *12 (10)* | *57 (9)* |  |
| *HIV-1 VL ≥1000* | *34 (6)* | *18 (15)* | *52 (8)* |  |
| *Missing HIV-1 VL* | *28 (5)* | *8 (7)* | *36 (5)* |  |
| Taking ART | 536 (100) | 120 (100) | 656 (100) |  |
| *NNRTI regimen (% of ART)* | *491 (92)* | *109 (91)* | *600 (92)* |  |
| *PI-based regimen* | *45 (8)* | *11 (9)* | *56 (8)* |  |
| *Current TDF Exposure* | *490 (69)* | *122 (65)* | *495 (76)* |  |
| *Current Abacavir Exposure* | *4 (1)* | *1 (1)* | *5 (1)* |  |
| Duration on ART (months) | 31 [15 - 59] | 33 [17 - 57] | 31 [15 - 58] |  |
| **Cardiovascular risk factors** | | | |  |
| BMI>30 kg/m2 | 102 (19) | 17 (14) | 119 (18) |  |
| Large Waist Circumference ^a^ | 264 (49) | 49 (41) | 313 (48) |  |
| Current smoker | 77 (14) | 11 (9) | 88 (13) |  |
| Diabetes Mellitus ^b^ | 14 (3) | 9 (8) | 26 (4) |  |
| Total cholesterol (mmol/L) | 4.40 [3.70 – 5.00] | 4.70 [4.10 – 5.58] | 4.40 [3.80 – 5.00] |  |
| LDL cholesterol (mmol/L) | 2.47 [1.92 – 2.92] | 2.68 [2.11 – 3.52] | 2.49 [1.95 – 3.02] |  |
| Hypertension ^c^ | 106 (20) | 45 (38) | 151 (23) |  |
| Family History | 28 (6) | 8 (7) | 36 (6) |  |
| Previous CVE | 12 (2) | 2 (2) | 14 (2) |  |
| **Laboratory values** | | | |  |
| ACR (mg/g) | 8.9 [6.0 – 13.8] | 63.6 [41.2 – 116.1] | 10.5 [6.6 – 22.4] |  |
| eGFR _CKD-EPI_ (mL/min/1.73m^2^) | 122.7 [107.7 – 133.4] | 112.8 [92.2 – 128.9] | 120.5 [104.2 – 132.4] |  |
| *eGFR< 60* | *1 (0)* | *7 (6)* | *8 (1)* |  |
| *eGFR 60-90* | *40 (8)* | *20 (17)* | *60 (9)* |  |
| *eGFR ≥ 90* | *495 (92)* | *93 (78)* | *588 (90)* |  |
| eGFR _MDRD_ (mL/min/1.73m^2^) | 114.7 [97.8 – 132.0] | 103.6 [86.2 – 123.6] | 113.1 [95.8 – 131.8] |  |
| *eGFR< 60* | *3 (1)* | *7 (6)* | *10 (2)* |  |
| *eGFR 60-90* | *61 (11)* | *28 (23)* | *89 (14)* |  |
| *eGFR ≥ 90* | *472 (88)* | *85 (71)* | *557 (85)* |  |
| Serum creatinine (umol/L) | 66 [57 - 75] | 69 [57 - 82] | 66 [57 - 76] |  |
| ALT (U/L) | 23 [17 - 32] | 23 [18 - 33] | 23 [17 - 32] |  |

**Legend for S1 Table:**

*p-values are significant (p<0.05); ^+^ p-values between 0.05 and 0.10. Data are given as number (%) or median [IQR].

^a^ Large waist circumference: >94 cm men or > 80 cm women; ^b^ Diabetes mellitus: HbA_1_c > 6.5% or use of diabetes medication; ^c^ Hypertension: Systolic blood pressure ≥ 140 mmHg, diastolic blood pressure ≥ 90 mmHg or use of antihypertensive medication. ACR = Albumine – Creatinine Ratio; ALT = alanine aminotransferase (mmol/l); ART = anti-retroviral treatment; BMI = Body Mass Index; CKD-EPI = Chronic Kidney Disease – Epidemiology; CVE = cardiovascular event; eGFR = estimated glomerular filtration rate; HIV = Human Immunodeficiency Virus; IQR = Inter-Quartile Range; LDL = Low-density lipoprotein; MDRD = Modification of Diet in Renal Disease; NNRTI = Non-nucleoside reverse-transcriptase inhibitors; PI-based = protease inhibitor-based; TDF = Tenofovir; VL = viral load.
