# Supplementary material for: Albuminuria Is Associated with Traditional Cardiovascular Risk Factors and Viral Load in HIV-Infected Patients in Rural South Africa
Source: PLoS One. 2015 Aug 26;10(8):e0136529. doi: 10.1371/journal.pone.0136529 (PMC4550462; doi:10.1371/journal.pone.0136529)
Supplement: S2 Table — (DOC) [file pone.0136529.s002.doc]

**S2 Table. Variables associated with albuminuria ^a^ in univariate and multivariate analysis in HIV-infected patients on ART treatment for > 6 months**

| **Variable** | **Univariate *p*-value** | **Multivariate aOR (95% CI)** | **Multivariate *p*-value** |
| --- | --- | --- | --- |
| **Demographic factors** | | | |
| Age (years) | 0.002* | 1.004 (0.977 – 1.032) | 0.76 |
| Gender Female | 0.15 | - | - |
| **HIV status** | | | |
| Months since positive HIV test | 0.71 | 0.999 (0.990 – 1.007) | 0.75 |
| Most recent CD4 cell count (cells/mm^3^) | 0.58 | 1.000 (0.999 – 1.001) | 0.88 |
| HIV-1 VL >50 copies/mL | 0.005 * | 2.736 (1.564 – 4.787) | <0.001 * |
| Taking ART | - | - | - |
| *NNRTI regimen (% of ART)* | *0.79* | *0.867 (0.378 – 1.986)* | *0.74* |
| *PI-based regimen* | *0.79* | *-* | *-* |
| *Current TDF Exposure* | *0.41* | *0.973 (0.552 – 1.715)* | *0.93* |
| *Current Abacavir Exposure* | *1.00* | *-* | *-* |
| Duration on ART (months) | 0.75 | Y | - |
| **Cardiovascular risk factors** | | | |
| BMI>30 kg/m2 | 0.21 | - | - |
| Large Waist Circumference ^b^ | 0.11 | - | - |
| Current smoker | 0.13 | - | - |
| Diabetes Mellitus ^c^ | 0.02* | 1.399 (0.518 – 3.778) | 0.51 |
| Total cholesterol (mmol/L) | <0.001* | 1.437 (1.163 – 1.776) | <0.001* |
| LDL cholesterol (mmol/L) | 0.003* | Y | - |
| Hypertension ^d^ | <0.001* | 1.895 (1.139 – 3.154) | 0.01* |
| Family History | 0.47 | - | - |
| Previous CVE | 1.00 | - | - |
| **Laboratory values** | | | |
| eGFR _CKD-EPI_ (mL/min/1.73m^2^) | <0.001* | 0.981 (0.970 – 0.992) | <0.001* |
| ALT (U/L) | 0.66 | - | - |

**Legend for S2 Table:**

*p-values are significant (p<0.05); ^+^ p-values between 0.05 and 0.10; ^Y^ Excluded due to collinearity with another variable.

Included subjects: 602. Number of subjects with albuminuria: 105.

^a^ Albuminuria: ACR>30 mg/g; ^b^ Large waist circumference: >94 cm men or > 80 cm women; ^c^ Diabetes mellitus: HbA_1_c > 6.5% or use of diabetes medication; ^d^ Hypertension: Systolic blood pressure ≥ 140 mmHg, diastolic blood pressure ≥ 90 mmHg or use of antihypertensive medication.

ACR = Albumine – Creatinine Ratio; ALT = alanine aminotransferase (mmol/l); aOR = adjusted odds ratio; ART = anti-retroviral treatment; BMI = Body Mass Index; 95% CI = 95% Confidence Interval; CKD-EPI = Chronic Kidney Disease – Epidemiology; CVE = cardiovascular event; eGFR = estimated glomerular filtration rate; HIV = Human Immunodeficiency Virus; LDL = Low-density lipoprotein; NNRTI = Non-nucleoside reverse-transcriptase inhibitors; PI-based = protease inhibitor-based; TDF = Tenofovir; VL = viral load.
