# Supplementary material for: Albuminuria Is Associated with Traditional Cardiovascular Risk Factors and Viral Load in HIV-Infected Patients in Rural South Africa
Source: PLoS One. 2015 Aug 26;10(8):e0136529. doi: 10.1371/journal.pone.0136529 (PMC4550462; doi:10.1371/journal.pone.0136529)
Supplement: S3 Table — (DOCX) [file pone.0136529.s003.docx]

**S3 Table. Characteristics of the patients with severely increased albuminuria (ACR> 300 mg/g)**

| **Variable** | **Total** |
| --- | --- |
| **Demographic factors** | |
| N (%) | 7 |
| Age in years | 40 [37 - 51] |
| Gender Female | 3 (43) |
| **HIV status** | |
| Months since positive HIV test | 19 [2 - 63] |
| Most recent CD4 cell count (cells/mm^3^) | 261 [40 - 458] |
| HIV-1 VL (copies/mL) |  |
| *HIV-1 VL <50* | *2 (29)* |
| *HIV-1 VL 50 - 999* | *0 (0)* |
| *HIV-1 VL ≥1000* | *2 (29)* |
| *Missing HIV-1 VL* | *3 (43)* |
| On ART | 7 (100) |
| *NNRTI regimen (% of patients on ART)* | *7 (100)* |
| *PI-based regimen* | *0 (0)* |
| *Current Tenofovir Exposure* | *6 (86)* |
| *Current Abacavir Exposure* | *0 (0)* |
| *Duration on ART in months* | *12 [0 - 18]* |
| **Cardiovascular risk factors** | |
| BMI>30 kg/m^2^ | 1 (14) |
| Large Waist Circumference ^a^ | 2 (29) |
| Current smoker | 1 (14) |
| Diabetes Mellitus ^b^ | 2 (29) |
| Total cholesterol (mmol/L) | 5.20 [4.40 – 6.70] |
| LDL cholesterol (mmol/L) | 3.16 [2.32 – 4.47] |
| Hypertension ^c^ | 3 (43) |
| Family History | 0 (0) |
| Previous CVE | 0 (0) |
| **Laboratory values** | |
| eGFR _CKD-EPI_ (mL/min/1.73m^2^) | 99.9 [88.8 – 131.6] |
| *eGFR< 60* | *0 (0)* |
| *eGFR 60-90* | *2 (29)* |
| *eGFR ≥ 90* | *5 (71)* |
| eGFR _MDRD_ (mL/min/1.73m^2^) | 89.9 [84.6 – 132.5] |
| *eGFR< 60* | *0 (0)* |
| *eGFR 60-90* | *4 (57)* |
| *eGFR ≥ 90* | *3 (43)* |
| Creatinine_serum_ (umol/L) | 76 [70 - 84] |
| ALT (U/L) | 24 [17 - 32] |

**Legend for S3 Table:**

Data are given as number (%) or median [Inter-Quartile Range].

^a^ Large waist circumference: >94 cm men or > 80 cm women; ^b^ Diabetes mellitus: HbA_1_c > 6.5% or use of diabetes medication; ^c^ Hypertension: Systolic blood pressure ≥ 140 mmHg, diastolic blood pressure ≥ 90 mmHg or use of antihypertensive medication.

ACR = Albumine – Creatinine Ratio; ALT = alanine aminotransferase (mmol/l); ART = anti-retroviral treatment; BMI = Body Mass Index; CVE = cardiovascular event; CKD-EPI = Chronic Kidney Disease – Epidemiology; eGFR = estimated glomerular filtration rate; HIV = Human Immunodeficiency Virus; LDL = Low-density lipoprotein; MDRD = Modification of Diet in Renal Disease; NNRTI = Non-nucleoside reverse-transcriptase inhibitors; PI-based = protease inhibitor-based; VL = viral load.
